# Comparative analysis of adiposity indices for predicting 2-year hypertension incidence in children and adolescents: a retrospective study

**DOI:** 10.1038/s41390-025-04155-1

**Published:** 2025-06-10

**Authors:** Bowen Zhu, Shumin Zhan, Hui Shi, Xingyun Wang, Jingwen Yue, Jianfang Gao, Tongshuai Wang, Rui Wang, Xirong Guo, Junfen Fu

**Affiliations:** 1 Department of Endocrinology, Children’s Hospital, Zhejiang University School of Medicine, National Clinical Research Center for Child Health, Hangzhou, China; 2 Hongqiao International Institute of Medicine, Tongren Hospital, Shanghai Jiao Tong University School of Medicine, Shanghai, China; 3 Xinhua Hospital, Shanghai Jiao Tong University School of Medicine, Shanghai, China

## Abstract

**Background:**

The aim of this study was to systemically examine the concomitant impact of adiposity indices on hypertension in children and adolescents.

**Methods:**

A community-based cohort study conducted by the China Health and Nutrition Survey (CHNS) 2009–2011 included 262 children and adolescents aged 7–17 years. Anthropometric and lipid profiles were analyzed for hypertension risk using logistic and cross-lagged models.

**Results:**

Among the 227 participants (mean age, 16.5 years), 147 (53.9%) were boys. Over 2 years, 26.4% of patients developed hypertension, 5.3% had elevated systolic blood pressure (SBP), and 25.6% had elevated diastolic blood pressure (DBP). After adjusting for covariates, higher body mass index (BMI), waist circumference (WC), hip circumference (HC), triceps skinfold thickness (TST), and body roundness index (BRI) were associated with an increased risk of hypertension. Incorporating these measures into a BP-based model improved the area under the curve (AUC) for hypertension prediction from 0.551 to 0.670. A longitudinal relationship was observed between adiposity indices and hypertension symptoms.

**Conclusions:**

This study suggests that anthropometric parameters are superior to blood lipid indicators in predicting the occurrence of hypertension in children. Abdominal obesity, as indicated by WC, HC, and BRI, accounts for a significant proportion of the risk of hypertension, particularly in children aged 7–12 years.

**Impact:**

- Anthropometric parameters are superior to blood lipid indicators in predicting the occurrence of hypertension in children.
- Abdominal obesity, as indicated by WC, and HC, accounts for a significant proportion of the risk of hypertension, particularly in children aged 7–12 years.
- The indices of BMI-WC-HC or BRI-HC appear to be the most promising indices for prevention and risk stratification for pediatric hypertension.

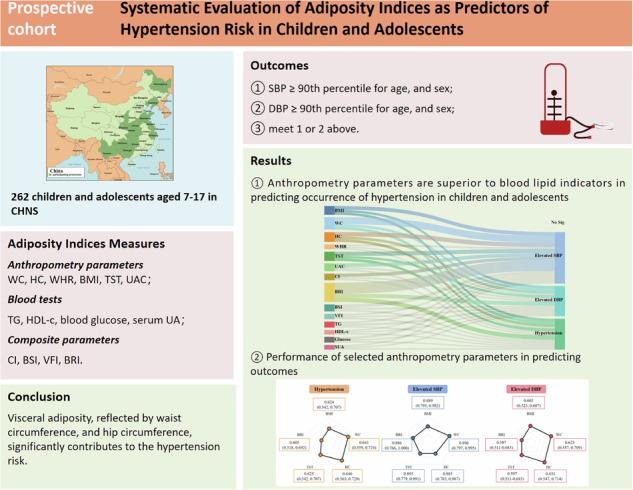

## Introduction

Hypertension affects approximately 4% of children and 11% of adolescents globally.^1^ Early identification and proper intervention for high blood pressure (HBP) or hypertension during childhood are crucial because these conditions are related to cardiovascular morbidity in adulthood and are commonly associated with obesity.^2–4^ A comprehensive analysis of 2033 studies spanning 154 countries revealed a significant prevalence of overweight and obesity among children and adolescents.^5^ Pooled estimates indicated that 8.5% of young individuals are overweight and 14.8% are obese, which translates to approximately one in every five young individuals facing the challenge of excess weight. China’s rapid economic progress has brought about major lifestyle and societal transformations, which have resulted in a surge in metabolic disorders among children.^6,7^ This trend is likely to have a substantial effect on the cardiovascular disease burden.^8^ The number of children aged 5–19 years with obesity is predicted to increase to 254 million by 2030.^9^ It is believed that obesity-linked primary hypertension has contributed to hypertension prevalence doubling from 1% to 4% over 30 years.^10,11^

Enhancing our understanding of adiposity profiles in the context of hypertension is essential for the prevention of this condition in children and adolescents. A cohort study involving young individuals found that high-normal body weight (exceeding the 60th percentile of age-specific body mass index (BMI)) was linked to a higher risk of hypertension.^12^ The suggested thresholds of 0.50 and 0.46 for two distinct pediatric groups have demonstrated robust predictive capabilities for identifying individuals with two or more cardiometabolic risk factors (CMRFs) in populations from six countries.^13^ Furthermore, innovative anthropometric indices, such as the body shape index (BSI), body roundness index (BRI), conicity index (CI), and visceral fat index (VFI), have been introduced to more accurately pinpoint subcutaneous and visceral fat distributions.^14^ A meta-analysis of 21,108 children and adolescents confirmed that the original BSI and modified BSI do not perform as well as traditional anthropometric indicators such as BMI and waist circumference (WC). BMI is the most effective indicator for screening HBP in pediatric populations.^15^ Chen et al. reported that the weight-to-height ratio (WHR) and BRI can be recommended to identify hypertension, dyslipidemia, abdominal obesity, and clustered CMRFs in 7–17-year-old teenagers.^16^ However, most studies have been conducted on adult populations, and the interplay and individual impact of biochemical measures, anthropometric data, and composite metrics on hypertension remains to be fully elucidated. Furthermore, data that can differentiate between hypertension risk in childhood associated with initial obesity-related metrics and predictive capacity are scarce. In the study, we aimed to systemically investigate the temporal and bidirectional relationship between anthropometric and biochemical measures of obesity and hypertension symptoms and compare the predictive ability of obesity-related measures for hypertension status using an observational cohort followed for 2 years.

## Materials and methods

### Study cohorts

This survey included children and adolescents aged 7–17 years with two follow-up visits conducted between 2009 and 2011. Valid blood pressure (BP) data, comprising three repeated readings, were obtained. Participants with missing BP values during the follow-up period were excluded from the analysis. The China Health and Nutrition Survey (CHNS) was a comprehensive nationwide prospective cohort study that included a representative sample of the Chinese population. Further details regarding the study design and data collection methodologies of the CHNS have been documented in prior publications.^17^ Blood samples were collected in 2009 and data information on biochemical indicators were obtained. Since the blood samples were collected in 2009, 768 participants with blood biomarker information were collected in 2009. After excluding those with 3-day high protein intake (*n* = 17), elevated systolic blood pressure (SBP) and diastolic blood pressure (DBP) (defined as age- and sex-specific 90th percentile, *n* = 221), glucose above 7.0 mmol/L or hemoglobin A1c (HbA1c) above 6.5% (*n* = 8), BMI above 35 kg/m^2^ (*n* = 1), missing data of SBP or DBP (*n* = 133), 388 participants were included in the formal analysis. A total of 161 participants were excluded because they were lost to follow-up (Supplemental Fig. S1). The demographic and behavioral characteristics of the participants included in the final analysis (*n* = 227) were compared with those of the excluded participants (Supplemental Table S1). All data and samples were collected after written informed consent was obtained from parents or caregivers of children and adolescents aged 7–17 years. The CHNS was approved by the Institutional Review Board of the University of North Carolina at Chapel Hill and by the local Institutional Review Board of the Ethics Committee.

### Data collection

A standardized structured questionnaire was administered by trained health staff to collect sociodemographic variables (in 2009), including age, sex, urban–rural residence, soft/sugared fruit drinks, total protein intake, total fat intake, total carbohydrate intake, and total energy intake. Physical examinations of WC, hip circumference (HC), height, weight, triceps skinfold thickness (TST), upper arm circumference (UAC), and BP were performed by a trained clinical staff. All individuals maintained a regular life pattern for at least 3 days before blood sample collection, and 12 mL of blood was collected (in three 4 mL tubes) on an empty stomach. Biomarker data collected in CHNS 2009 involved the release of 26 fasting blood measurements in individuals aged 7 years and older.^18^ The estimated glomerular filtration rate (eGFR) was calculated using the Schwartz formula.^19^ Individuals were categorized into two groups: residential, urban, and rural. Individual dietary intake for 3 consecutive days was determined for each household member. This step was achieved by asking individuals each day to report all food consumed away from home on a 24-h recall basis, and the same daily interview was used to collect at-home individual consumption. Soft/sugared fruit drink information was assessed using questions, including “Drink soft/sugared fruit drinks?” or “How often drink soft/sugared fruit drinks?”

### Adiposity index measures

Adiposity index measurements included BMI, WC, HC, TST, waist-to-hip circumference ratio (WHR), UAC, triglycerides (TG), high-density lipoprotein cholesterol (HDL-c), blood glucose, serum uric acid (SUA), CI, BSI, VFI, and BRI. BMI was calculated as weight (kg)/height^2^ (m^2^) and converted into age- and sex-specific BMI percentiles.^20^ Abdominal obesity was defined as WC age- and sex-specific 90th percentile, determined by the cut-off points of the standards for Chinese children and adolescents.^21^ WHR was calculated as WC (cm)/height (cm). The calculation of the novel anthropometric indices were described below, with relevant references cited in parentheses (CI^22^, BSI^23^, VFI^24^, BRI^25^):

$${{{\rm{C}}}}{{{\rm{I}}}}={{{\rm{W}}}}{{{\rm{C}}}}/0.109^{\ast} {({{{\rm{W}}}}{{{\rm{t}}}}/{{{\rm{H}}}}{{{\rm{t}}}})}^{1/2}$$

$${{{\rm{B}}}}{{{\rm{S}}}}{{{\rm{I}}}}={{{\rm{W}}}}{{{\rm{C}}}}\ast {{{{\rm{W}}}}{{{\rm{t}}}}}^{-2/3}\ast {{{{\rm{H}}}}{{{\rm{t}}}}}^{-5/6}$$

$${{{\rm{V}}}}{{{\rm{F}}}}{{{\rm{I}}}}={{{\rm{W}}}}{{{\rm{C}}}}/[39.68+1.88^{\ast} {{{\rm{B}}}}{{{\rm{M}}}}{{{\rm{I}}}}^{\ast} {{{\rm{T}}}}{{{\rm{G}}}}/1.03]{*} [1.31/{{{\rm{H}}}}{{{\rm{D}}}}{{{\rm{L}}}}{\textstyle {\mbox{-}}}{{{\rm{c}}}}]\,({{{\rm{f}}}}{{{\rm{o}}}}{{{\rm{r}}}}\,{{{\rm{m}}}}{{{\rm{a}}}}{{{\rm{l}}}}{{{\rm{e}}}})$$

$${{{\rm{V}}}}{{{\rm{F}}}}{{{\rm{I}}}}={{{\rm{W}}}}{{{\rm{C}}}}/(36.\,58+1.89^{\ast} {{{\rm{B}}}}{{{\rm{M}}}}{{{\rm{I}}}})^{\ast} ({{{\rm{T}}}}{{{\rm{G}}}}/0.\,81)^{\ast} (1.\,52/{{{\rm{H}}}}{{{\rm{D}}}}{{{\rm{L}}}}{\textstyle {\mbox{-}}}{{{\rm{c}}}})\,({{{\rm{f}}}}{{{\rm{o}}}}{{{\rm{r}}}}\,{{{\rm{f}}}}{{{\rm{e}}}}{{{\rm{m}}}}{{{\rm{a}}}}{{{\rm{l}}}}{{{\rm{e}}}})$$

$${{{\rm{BRI}}}}=364.2-365.5* {(1-((0.5* {{{\rm{WC}}}}/{{{\rm{\pi }}}})2/(0.5* {{{\rm{Ht}}}})2))}^{0.5}$$

The units of TG, HDL-c, and LDL-c are mmol/L.

TG levels were measured using GPO-PAP (Hitachi 7600, Kyowa, Japan). HDL-c levels were measured using an enzymatic method (Hitachi 7600, Kyowa, UK). Glucose levels were measured using GOD-PAP (Hitachi 7600, Randox, UK).

### Diagnostic criteria and definitions of outcome

SBP or DBP values were determined based on the average of three separate measurements. Participants’ BP status, classified as either normal or elevated, was ascertained using sex- and age-specific cut-off values that apply to Chinese school-age children and adolescents. According to Cook’s criteria, elevated BP is defined as SBP or DBP equal to or greater than the 90th percentile for age, sex, and height.^26^ BP outcomes were evaluated using three distinct categorization methods: hypertension (defined by elevated SBP or DBP) versus non-hypertension, elevated SBP versus normal SBP, and elevated DBP versus normal DBP.

### Statistical analysis

Data are presented as mean ± standard deviation (SD) and median with interquartile range for continuous variables or number (percentage) for categorical variables. Demographic, anthropometric, and biochemical data were compared using the chi-square test and Fisher’s exact test for categorical variables. The Wilcoxon rank-sum test was used to analyze continuous variables. Multivariable logistic regression models were used to evaluate the longitudinal associations between adiposity indices and incident hypertension, elevated SBP, and elevated DBP. Multivariable models were sequentially adjusted for (1) SBP and/or DBP; (2) age and sex; and (3) baseline residence, eGFR, soft fruit drinks, and total carbohydrate intake. The area under the receiver operating characteristic curve (AUC) was calculated to assess the predictive ability of adiposity indices for hypertension, elevated SBP, and elevated DBP. Continuous net reclassification improvement and absolute integrated discrimination improvement were used to assess whether adding obesity-related measures could improve risk discrimination and reclassification for hypertension, elevated SBP, and elevated DBP predictions above baseline SBP and/or DBP. Cross-lagged panel models were applied to analyze the temporal relationship between obesity-related measures including BMI, CI, WC, HC, TST, UAC, and BRI (as continuous variables) at each phase and elevated SBP and/or DBP levels (as categorical variables). The cross-lagged panel model is a structural model that calculates reciprocal and longitudinal relationships among the variables.^27^ Cross-lagged models were conducted using SAS software version 9.4 (Proc calis procedure). To identify causality, all autoregressive and cross-lagged paths were included in the Model. In addition to the basic model with obesity-related measures and SBP and/or DBP, the model paths include: (1) paths from obesity-related measures to elevated SBP and/or DBP levels; (2) paths from elevated SBP and/or DBP levels to obesity-related measures. All anthropometric indices (BMI, CI, WC, HC, TST, UAC, CI, VFI, BSI, and BRI) and metabolic measures (TG, HDL-c, Glucose, SUA) were analyzed as continuous variables, while the outcome of hypertension was analyzed as categorical variable Several sensitivity analyses were conducted to test the robustness of the findings. First, we treated obesity-related measures, SBP, and DBP as continuous variables to determine linear associations between adiposity indices and BP. Pre-specified subgroup analyses were conducted based on age (7–12 years and 13–17 years) and sex (boys and girls) in the secondary analyses. *P* < 0.05 (two-sided) was considered statistically significant. All analyses were conducted using the SAS software (v.9.4, SAS Institute Inc., Cary, NC).

## Result

### Baseline characteristics of pediatric and adolescent participants across SBP and DBP groups

Among the 227 participants included in the present analysis (64.8% of whom were boys), the mean age was 11.7 (SD, ±2.3) years. During a median follow-up period of 2 years, 60 cases of incident hypertension (26.4%), 12 cases of incident elevated SBP (5.3%), and 58 cases of elevated DBP (25.6%) were identified. Children diagnosed with hypertension had significantly higher WC, HC, BMI, TST, and BRI levels. Additionally, a higher proportion of these children were girls and classified as overweight. Compared with the normal SBP group, the children and adolescents diagnosed with elevated SBP had a higher WC, HC, BMI, TST, BRI, ferritin, and total protein, as well as higher proportions of overweight. Compared with the normal DBP group, the children and adolescents diagnosed with elevated DBP had a higher WC, HC, BMI, TST, low total carbohydrate intake, and a higher proportion of girls who were overweight (Table 1).

**Table 1:** Baseline characteristics of the individuals stratified by hypertension, systolic BP and diastolic BP in the CHNS dataset (*n* = 262)

	Hypertension	Non-hypertension	*P* value	Systolic BP >P 90	Systolic BP ≤P 90	*P* value	Diastolic BP >P 90	Diastolic BP ≤P 90	*P* value
Participants (*n*)	60	167		12	215		58	169	
Demographics									
Age (years)	12.0 (2.4)	11.6 (2.3)	0.301	12.7 (2.7)	11.7 (2.3)	0.151	12 (2.5)	11.7 (2.2)	0.380
Boys (%)	28 (46.7)	119 (71.3)	<0.001	9 (75.0)	138 (64.2)	0.651	26 (44.8)	121 (71.6)	<0.001
Rural (%)	43 (71.7)	132 (79.0)	0.244	7 (58.3)	168 (78.1)	0.217	41 (70.7)	134 (79.3)	0.177
Anthropometry parameters									
WC (cm)	68.9 (11.4)	63.8 (9.7)	0.001	79.7 (13.7)	64.4 (9.5)	0.003	68.3 (11.1)	64.1 (9.9)	0.009
HC (cm)	82.0 (10.5)	76.8 (11.1)	0.002	90.1 (11.1)	77.5 (10.8)	<0.001	81.5 (10.2)	77 (11.3)	0.009
WHR	0.84 (0.10)	0.83 (0.08)	0.307	0.84 (0.09)	0.83 (0.09)	0.730	0.84 (0.10)	0.83 (0.08)	0.495
BMI (kg/m^2^)	18.2 (4.0)	16.7 (3.0)	0.008	20.9 (5.0)	16.9 (3.1)	<0.001	18.0 (4.0)	16.8 (3.0)	0.028
Overweight (>P 75)	10 (16.7)	15 (9.0)	0.103	5 (41.7)	20 (9.3)	<0.001	9 (15.5)	16 (9.5)	0.204
Systolic BP (mm Hg)	95.5 (13.4)	93.3 (10.7)	0.250	109.0 (8.7)	93.1 (11.0)	<0.001	94.8 (13.0)	93.6 (11.0)	0.496
Diastolic BP (mm Hg)	61.5 (8.0)	61 (6.7)	0.647	68.1 (5.3)	60.7 (7.0)	<0.001	61.1 (7.9)	61.1 (6.8)	0.980
Triceps skinfold thickness (TST, cm)	1.28 (0.93–1.73)	1.07 (0.73–1.53)	0.008	1.95 (0.97–3.7)	1.10 (0.80–1.57)	0.015	1.22 (0.93–1.70)	1.1 (0.73–1.57)	0.030
UAC (cm)	22 (4.5)	21 (4.4)	0.095	26 (4.8)	21 (4.3)	<0.001	22 (4.3)	21 (4.5)	0.283
Blood tests									
Albumin, g/L	48.6 (3.1)	48.2 (3.2)	0.456	49.8 (3.6)	48.2 (3.1)	0.095	48.5 (2.9)	48.2 (3.3)	0.539
Alanine transaminase, U/L	13.0 (10.5–15.0)	14.0 (11.0–18.0)	0.286	13.5 (11.5–25.0)	14.0 (10.0–17.0)	0.271	13.0 (10.0–15.0)	14.0 (11.0–18.0)	0.183
Apolipoprotein B, mg/dL	0.7 (0.5–0.8)	0.6 (0.5–0.7)	0.142	0.7 (0.5–0.9)	0.6 (0.5–0.7)	0.212	0.6 (0.5–0.8)	0.6 (0.5–0.7)	0.287
Apolipoprotein A-1, mg/dL	0.96 (0.85–1.09)	0.98 (0.87–1.09)	0.558	1.04 (0.96–1.10)	0.97 (0.86–1.09)	0.196	0.95 (0.84–1.06)	0.98 (0.87–1.09)	0.339
Ferritin, ng/mL	43.4 (28.6–65.1)	43.9 (28.4–58.0)	0.591	64.3 (45.6–78.0)	42.8 (28.4–58.1)	0.048	42.7 (28.6–63.0)	43.9 (28.8–58.2)	0.866
Serum magnesium, mmol/L	0.94 (0.09)	0.95 (0.07)	0.424	0.93 (0.12)	0.95 (0.07)	0.579	0.95 (0.09)	0.95 (0.07)	0.569
Transferrin, mg/gL	287.5 (264.5–329.0)	295.0 (265.0–325.0)	0.972	299.0 (269.0–321.5)	293.0 (265.0–325.0)	0.871	287.5 (265.0–330.0)	295.0 (265.0–325.0)	0.900
Soluble transferrin receptor, mg/L	1.5 (1.3–2.0)	1.5 (1.3–1.9)	0.654	1.5 (1.3–2.0)	1.5 (1.3–1.9)	0.977	1.5 (1.3–1.9)	1.5 (1.3–1.9)	0.675
Total protein, g/L	76.6 (5.3)	76.2 (4.8)	0.570	79.1 (5.2)	76.1 (4.9)	0.040	76.5 (5.3)	76.2 (4.8)	0.738
Serum uric acid, mg/dL	5.2 (1.5)	5.1 (1.3)	0.801	5.7 (1.6)	5.1 (1.3)	0.108	5.2 (1.5)	5.1 (1.3)	0.937
Total cholesterol, mmol/L	4.0 (0.7)	3.8 (0.6)	0.035	4.0 (0.8)	3.8 (0.6)	0.298	4 (0.6)	3.8 (0.6)	0.068
HDL cholesterol, mmol/L	1.4 (1.2–1.7)	1.4 (1.2–1.6)	0.771	1.4 (1.3–1.6)	1.4 (1.2–1.6)	0.678	1.3 (1.2–1.6)	1.4 (1.2–1.6)	0.613
LDL cholesterol, mmol/L	2.2 (1.8–2.6)	2.2 (1.7–2.6)	0.249	2.2 (1.7–2.8)	2.2 (1.8–2.6)	0.708	2.2 (1.8–2.6)	2.2 (1.8–2.6)	0.344
Triglycerides, mg/dL	0.8 (0.5–1.3)	0.7 (0.5–1.1)	0.745	0.8 (0.5–1.3)	0.7 (0.5–1.2)	0.759	0.8 (0.5–1.2)	0.7 (0.5–1.1)	0.939
eGFR, ml/ min/l.73 m^2^	82.4 (9.2)	81.3 (11.6)	0.481	81.6 (6.8)	81.6 (11.1)	0.991	82.6 (9.2)	81.3 (11.6)	0.427
HbA1c, %	5.3 (0.4)	5.3 (0.4)	0.907	5.2 (0.5)	5.3 (0.4)	0.768	5.3 (0.4)	5.3 (0.4)	0.862
Blood glucose, mmol/L	4.8 (0.5)	4.9 (0.5)	0.729	5.1 (0.5)	4.8 (0.5)	0.075	4.8 (0.5)	4.9 (0.5)	0.442
Creatinine, mg/dL	67.0 (62.0–75.0)	67.0 (61.0–73.0)	0.651	70.0 (63.5–76.0)	67.0 (61.0–73.0)	0.177	66.5 (62.0–74.0)	67.0 (61.0–73.0)	0.917
Composite parameters									
CI	1.15 (1.07–1.23)	1.13 (1.08–1.19)	0.427	1.20 (1.11–1.31)	1.13 (1.08–1.19)	0.058	1.14 (1.07–1.25)	1.13 (1.08–1.19)	0.559
BSI	0.08 (0.08–0.09)	0.08 (0.08–0.08)	0.432	0.08 (0.08–0.09)	0.08 (0.08–0.08)	0.399	0.08 (0.07–0.09)	0.08 (0.08–0.08)	0.449
VFI	0.92 (0.48–1.44)	0.70 (0.43–1.15)	0.139	0.77 (0.51–1.42)	0.74 (0.43–1.25)	0.650	0.92 (0.48–1.43)	0.7 (0.43–1.16)	0.186
BRI	2.26 (1.67–3.31)	1.95 (1.53–2.4)	0.025	3.55 (1.68–5.17)	1.97 (1.53–2.47)	0.021	2.19 (1.59–3.20)	1.95 (1.53–2.42)	0.061
Health-related behavior									
Soft/sugared fruit drinks	54 (90.0)	146 (88.0)	0.670	12 (100.0)	188 (87.9)	0.369	52 (89.7)	148 (88.1)	0.748
Total protein intake (g/day)	49.8 (40.5–60.7)	50.2 (39.4–67.2)	0.530	50.8 (42.7–69.4)	50.1 (39.2–64.6)	0.338	49.8 (40.5–60.5)	50.2 (39.7–67.2)	0.433
Total fat intake (g/day)	55.1 (34.7–72.2)	53.3 (36.9–71.3)	0.830	1710.0 (1395.0–2078.9)	1645.4 (1324.6–1937.6)	0.205	1621.7 (1240.7–1928.7)	1670.9 (1357.8–1947.7)	0.930
Total carbohydrate intake (g/day)	218.9 (174.8–260.4)	235.7 (198.8–276.9)	0.076	218.9 (149.8–327.4)	234.3 (192.3–270.2)	0.563	218.9 (174.8–259.2)	235.7 (199.5–276.9)	0.045
Total energy intake (kcal/day)	1621.7 (1240.7–1933.9)	1670.9 (1353.3–1947.7)	0.317	61.8 (51.0–67.2)	52.4 (35.6–71.7)	0.641	51.0 (34.7–72.2)	53.4 (36.9–70.2)	0.218

Data are presented as No. (%), mean ± SD; *P* values were calculated by using the *T* test or the Wilcoxon test for continuous variables and the *χ*^2^ test or the Fisher exact test for categorical variables.

*BMI* body mass index, *BSI* body shape index, *BRI* body roundness index, *CI* conicity index, *DBP* diastolic blood pressure, *eGFR* estimated glomerular filtration rate, *HC* hip circumference, *HDL-c* high-density lipoprotein cholesterol, *HbA1c* hemoglobin A1C, *LDL-c* low-density lipoprotein cholesterol, *SBP* systolic blood pressure, *UAC* upper arm circumference, *VFI* visceral fat index, *WC* waist circumference, *WHR* waist-to-hip circumference ratio.

### Correlations between body adiposity measures and the onset of hypertension

**Fig. 1:**
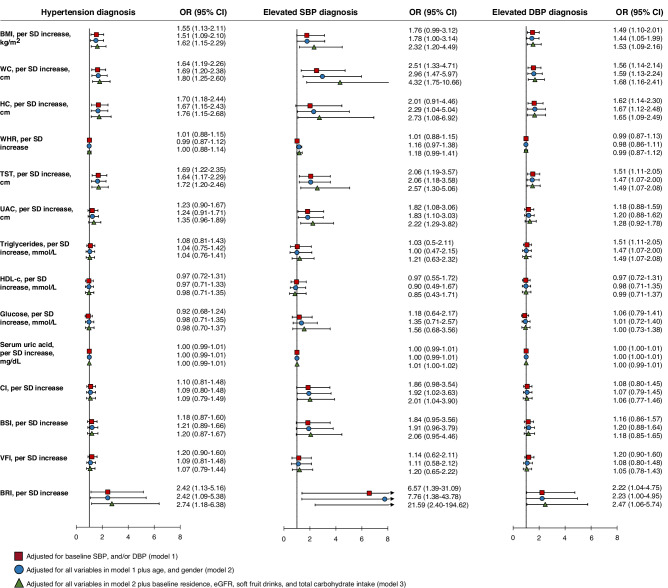
Associations between adiposity indices and incident hypertension. BMI body mass index, BRI body roundness index, BSI body shape index, CI conicity index, HDL high-density lipoprotein, SUA serum uric acid, TST triceps skinfold thickness, VFI visceral fat index, WHR waist-to-hip ratio.

The odds ratios and 95% confidence intervals (CIs) of incident hypertension for each adiposity index (per SD) are shown in Fig. 1. After adjusting for covariates, BMI, WC, HC, TST, and BRI were associated with a higher risk of hypertension. When defining the outcome as elevated SBP (equal to or more than 90th percentile for age, sex, and height), BMI, WC, HC, TST, UAC, SUA, CI, and BRI were associated with a higher risk of elevated SBP. BMI, WC, HC, TST, TG level, and BRI were associated with a higher risk for elevated DBP (Fig. 1).

### The incremental predictive value of adiposity indices in assessing the risk of hypertension, high SBP, and high DBP

Seven adiposity indices were identified: BMI, WC, HC, TST, UAC, BRI, and CI. The inclusion of HC and BRI enhanced the net reclassification ability and the overall predicted probability of hypertension. Similarly, incorporating WC was shown to improve the net reclassification ability and the overall predicted probability of elevated SBP. The addition of WC, HC, and BRI improved the net reclassification ability and the overall predicted probability of elevated DBP. Furthermore, by incorporating BMI, WC, and HC into the basic model, which considered continuous SBP and DBP, the AUC for hypertension prediction was significantly enhanced, increasing from 0.551 (95% CI: 0.463–0.640) to 0.670 (95% CI: 0.592–0.748). For SBP prediction, the AUC remained high at 0.873, with a CI ranging from 0.771 to 0.974. Similarly, for elevated DBP prediction, the AUC improved from 0.531 (95% CI: 0.440–0.621) to 0.649 (95% CI: 0.570–0.729) (Table 2).

**Table 2:** Performance of risk prediction models for 2-year risk of incident hypertension by including selected anthropometry parameters and innovative indices.

	AUC (95% CI)	Difference (95% CI)	NRI (95% CI)	Absolute IDI (95% CI)
2-Year diagnosis of hypertension				
Basic model^a^	0.551 (0.463, 0.640)	Reference	Reference	Reference
Basic model+BMI	0.624 (0.542, 0.707)	0.073 (−0.014, 0.161)	0.165 (−0.120, 0.451)	0.036 (0.005, 0.066)*
Basic model+WC	0.641 (0.559, 0.724)	0.090 (−0.012, 0.192)	0.244 (−0.044, 0.531)	0.044 (0.013, 0.075)**
Basic model+HC	0.646 (0.563, 0.729)	0.095 (−0.001, 0.190)	0.468 (0.189, 0.748)*	0.040 (0.013, 0.066)**
Basic model+TST	0.625 (0.542, 0.707)	0.073 (−0.029, 0.176)	0.148 (0.076, 0.658)*	0.027 (−0.009, 0.063)
Basic model+BRI	0.605 (0.518, 0.692)	0.054 (−0.048, 0.156)	0.360 (0.069, 0.651)*	0.041 (0.007, 0.074)*
Basic model+BRI, HC	0.655 (0.574, 0.735)	0.103 (0.003, 0.204)*	0.527 (0.241, 0.812)*	0.056 (0.020, 0.092)**
Basic model+BMI, WC, HC	0.670 (0.592, 0.748)	0.119 (0.021, 0.217)*	0.455 (0.168, 0.742)**	0.051 (0.018, 0.084)**
2-Year diagnosis of elevated SBP				
Basic model^b^	0.873 (0.771, 0.974)	Reference	Reference	Reference
Basic model+BMI	0.889 (0.795, 0.982)	0.016 (−0.026, 0.058)	0.243 (−0.324, 0.818)	0.051 (0.034, 0.137)
Basic model+WC	0.896 (0.797, 0.995)	0.023 (−0.036, 0.082)	0.700 (0.153, 1.249)*	0.113 (0.001, 0.225)*
Basic model+HC	0.885 (0.783, 0.987)	0.013 (−0.029, 0.054)	0.209 (−0.365, 0.782)	0.037 (−0.02, 0.094)
Basic model+TST	0.885 (0.779, 0.991)	0.013 (−0.048, 0.073)	0.413 (−0.160, 0.986)	0.144 (−0.031, 0.318)
Basic model+UAC	0.888 (0.801, 0.975)	0.015 (−0.026, 0.057)	0.784 (0.277, 1.290)*	0.048 (−0.018, 0.114)
Basic model+CI	0.888 (0.777, 0.999)	0.017 (−0.030, 0.063)	0.340 (−0.240, 0.919)	0.038 (−0.030, 0.105)
Basic model+BRI	0.886 (0.766, 1.000)	0.014 (−0.049, 0.077)	0.711 (0.142, 1.280)*	0.158 (−0.011, 0.328)
Basic model+BRI, HC	0.893 (0.776, 1.000)	0.021 (−0.039, 0.081)	0.636 (0.066, 1.207)*	0.157 (−0.01, 0.325)
Basic model+BMI, WC, HC	0.897 (0.797, 0.996)	0.024 (−0.034, 0.083)	0.710 (0.163, 1.258)*	0.113 (0.001, 0.224)*
2-Year diagnosis of elevated DBP				
Basic model^c^	0.531 (0.440, 0.621)	Reference	Reference	Reference
Basic model+BMI	0.605 (0.523, 0.687)	0.108 (−0.027, 0.242)	0.200 (−0.089, 0.490)	0.044 (0.015, 0.072)**
Basic model+WC	0.623 (0.537, 0.709)	0.125 (−0.012, 0.263)	0.333 (0.039, 0.628)*	0.037 (0.009, 0.065)**
Basic model+HC	0.631 (0.547, 0.714)	0.134 (0.008, 0.259)*	0.456 (0.175, 0.738)**	0.043 (0.014, 0.072)**
Basic model+TST	0.597 (0.511, 0.683)	0.100 (−0.042, −0.242)	0.322 (0.027, 0.618)*	0.013 (−0.021, −0.046)
Basic model+BRI	0.575 (0.480, 0.670)	0.078 (−0.072, 0.228)	0.349 (0.054, 0.643)*	0.052 (0.017, 0.087)**
Basic model+BRI, HC	0.639 (0.556, 0.721)	0.141 (0.006, 0.277)*	0.577 (0.292, 0.861)**	0.037 (0.009, 0.065)**
Basic model+BMI, WC, HC	0.649 (0.570, 0.729)	0.152 (0.022, 0.282)*	0.349 (0.054, 0.643)**	0.044 (0.014, 0.073)**

*AUC* area under the curve, *BMI* body mass index, *BRI* body roundness index, *CI* conicity index, *DBP* diastolic blood pressure, *HC* hip circumference, *SBP* systolic blood pressure, *TST* triceps skinfold thickness, *UAC* upper arm circumference, *WC* waist circumference.

*0.01 < *P* ≤ 0.05; **0.001 < *P* ≤ 0.01; ***P ≤ 0.001.

^a^Basic model: baseline age, gender, systolic BP, diastolic BP, and eGFR.

^b^Basic model: baseline age, gender, systolic BP, and eGFR.

^c^Basic model: baseline age, gender, and diastolic BP.

### The temporal dynamics of the association between body adiposity measures and elevated SBP/DBP

**Fig. 2:**
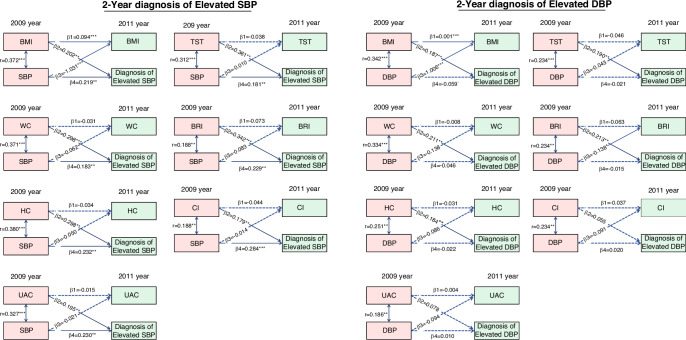
Estimates from the cross-lagged model for the associations between anthropometry parameters and incident hypertension classified by elevated SBP and elevated DBP. BMI body mass index, BRI body roundness index, CI conicity index, DBP diastolic blood pressure, HC hip circumference, SBP systolic blood pressure, TST triceps skinfold thickness, UAC upper arm circumference, WC waist circumference. All estimates were standardized. *0.01 < *P* ≤ 0.05; **0.001 < *P* ≤ 0.01; ***P < 0.001.

The fully cross-lagged model was used to compare both the adiposity index measures to BP levels (defined as elevated SBP or DBP) and BP levels to obesity-related measures (Fig. 2). Notably, there were no directional associations between most obesity-related measures and hypertension symptoms. Baseline adiposity indices including WC, HC, TST, UAC, BRI, and CI were unidirectionally associated with hypertension symptoms (elevated SBP or DBP). Cross-lagged path estimates showed that only BMI was directly and longitudinally associated with hypertension. Baseline SBP (in 2009) was negatively associated with later BMI (in 2011), whereas baseline DBP (in 2009) was significantly positively associated with BMI (in 2011).

### Sensitivity and subgroup analysis

In the sensitivity analysis, the linear analysis revealed significant longitudinal associations between various adiposity indices, including BMI, WC, HC, TST, UAC, BSI, and BRI, and a higher risk of SBP and DBP, which were treated as continuous variables (Supplemental Fig. S2). In the subgroup analysis, the significant associations of BMI, WC, HC, TST, and BRI with the risk of hypertension only existed in the younger age group (7–12 years) after accounting for multiple variables. In the subgroup classified by sex, a significant association between BMI and the risk of hypertension was observed only in girls. However, the association of UAC and high TG levels with the risk of hypertension existed only in boys (Supplemental Tables S2 and S3).

## Discussion

In this cohort study of Chinese children and adolescents, we observed a significant association between BMI, WC, HC, TST, and BRI, and incident hypertension. This association remained consistent regardless of initial SBP or DBP measurements, demographic variables, lifestyle factors, and eGFR. The relationships between these adiposity indices and both elevated SBP and DBP mirrored their associations with hypertension, except that increased UAC and CI were linked to a higher probability of elevated SBP. It is noteworthy that among the five adiposity indices, only BMI demonstrated a consistent, directional, and longitudinal association with the symptoms of hypertension.

Previous studies have consistently demonstrated a significant association between adiposity indicators and hypertension in pediatric populations.^12,28^ Our study adds to the existing literature by providing evidence that increased WC is positively correlated with elevated BP in the pediatric population. The accumulation of abdominal fat is known to secrete various adipokines and cytokines, which can lead to endothelial dysfunction and increased peripheral vascular resistance, thereby contributing to the development of hypertension.^29–31^ Moreover, our study revealed that expanded HC was associated with a higher degree of fat deposition in the buttocks and thighs, consequently increasing the likelihood of hypertension in the pediatric population. TST could serve as a valuable anthropometric marker for assessing cardiovascular risk in children. This association may be attributed to the secretion of adipose-derived hormones and inflammatory cytokines that can lead to endothelial dysfunction and heightened vascular resistance.^32^ The AUCs for WC and HC in predicting pediatric hypertension were higher than those for BMI. These findings suggest that body fat distribution, rather than overall obesity, may be a more sensitive predictor of cardiovascular health in the pediatric population.^33^

The AUC for WC and HC in the prediction of pediatric hypertension was notably higher than that for BMI. These results suggest that the distribution of body fat, rather than overall adiposity, may be a more sensitive predictor of cardiovascular health in the pediatric population. The BRI, which quantifies visceral fat and total body fat percentage, is significantly associated with elevated BP in children.^25^

Evidence suggests BRI serves as a more robust clinical predictor than standard anthropometric parameters for several adult-onset conditions, spanning cardiometabolic disorders, renal impairment, and malignancy risk.^34–38^ There is an emerging consensus that visceral fat poses a significantly greater health risk than subcutaneous fat, as it is more strongly associated with an increased risk of various diseases in adults.^39,40^ While the concept that BRI can estimate total and regional fat percentages seems plausible and potentially superior in adults, evidence linking BRI to diseases in children is limited. In this study, the AUC for BRI in predicting pediatric hypertension was found to be lower than that for WC and HC. This suggests that BRI’s predictive power of BRI for pediatric hypertension may not be as effective as it is in adults.

Theoretically, assuming the shape of the body to be an ellipse with a long axis height and a short axis WC, the BRI can be calculated as the eccentricity of the ellipse via human modeling. Acknowledging the inherent disparities in body contours between pediatric and adult demographics, it is crucial to appreciate that indices, such as the BRI, may not be directly comparable across these groups. The efficacy of the BRI as a predictor of hypertension could be subject to variation owing to the unique growth trajectories, body composition differences, and influence of developmental milestones that are characteristic of the pediatric population. Therefore, the application of the BRI in pediatric assessments should be approached with caution, and further research is needed to establish its validity and reliability in predicting hypertension, specifically within the pediatric demographic. For practical reasons, there remains a need for a simple and effective child-specific indicator that better reflects visceral obesity. Our study revealed that UAC and CI were significantly correlated with SBP but not with DBP. This distinction could be due to the different physiological mechanisms underlying the two types of BP. SBP is influenced more by arterial stiffness, cardiac output, and abnormal changes in brain cortical structure, whereas DBP is affected more by peripheral vascular resistance.^41,42^ The fact that our measurements were more strongly associated with SBP may indicate a closer link between the factors affecting arterial stiffness and brain cortical structure.

Our study findings also found that there is no association between blood lipid levels (TG, LDL-c, HDL-c, and SUA) and the development of hypertension. Our results contrast with established findings in adult populations, where both conventional lipid parameters and novel lipid-derived indices have been consistently shown to predict hypertension risk.^43,44^ The lack of association in our pediatric cohort may be explained by multiple factors. First, the pathophysiology of hypertension in children may differ fundamentally from that in adults, where lipid metabolism plays a more well-established role.^45^ Children are in a dynamic phase of growth and development, with a rapidly changing metabolic profile that may not yet be fully aligned with the traditional risk factors observed in adulthood.^46^

Additionally, the dietary and lifestyle factors that significantly influence lipid profiles in adults may not have had sufficient time to exert their effects in children. As children’s dietary habits and physical activity levels evolve, their lipid profiles may not stabilize to the point where they can significantly influence BP. This finding may also explain why lipid-related composite indices such as BSI and VFI cannot predict hypertension onset in children as effectively as they do in adult populations. The associations between adiposity indices and hypertension in children appear to be age-dependent, with our study indicating a significant correlation within the 7–12-year age group, which was not observed in the 13–18-year age group. This age-specific association may reflect the dynamic changes in growth, development, and metabolic patterns that occur during childhood and adolescence. Physiological changes during adolescence, including growth spurts, hormonal fluctuations, and maturation of various organ systems, could influence the relationship between adiposity indices and BP in complex ways that are not yet fully understood.

### Strengths and limitations

A significant advantage of our study is its community-based approach, which successfully enrolled a substantial number of children and adolescents aged 7–17 years in China. This age group has been notably underrepresented in research on pediatric hypertension. Limitations include the possibility of residual confounding inherent to the observational design, including the possibility of differential distribution of unmeasured or incompletely measured confounders such as birth weight and gestational age. We cannot exclude limitations in the generalizability of the results in this CHNS cohort with access to healthcare and the existence of withdrawal bias. Moreover, different criteria for recognizing overweight and obesity in children may have influenced the accuracy of the estimates.

### Conclusions and implications

This study is the first to compare the ability of adiposity indices for hypertension: BMI, WC, HC, TST, and BRI, for predicting the 2-year hypertension among Chinese children and adolescents.

Notably, only BMI demonstrated a consistent directional relationship with the longitudinal presentation of hypertension. Moreover, pronounced associations between these adiposity indices and the risk of hypertension were predominantly observed in the younger age group (7–12 years). The combined prediction model of BMI-WC-HC and BRI-HC appears to be the most promising index for prevention and risk stratification for pediatric hypertension.

## Supplementary information

Supplementary information

## Data Availability

Original data generated and analyzed during this study are included in this published article or the data repositories listed in the references.
