# Correction: Comparative analysis of adiposity indices for predicting 2-year hypertension incidence in children and adolescents: a retrospective study

**DOI:** 10.1038/s41390-025-04255-y

**Published:** 2025-07-11

**Authors:** Bowen Zhu, Shumin Zhan, Hui Shi, Xingyun Wang, Jingwen Yue, Jianfang Gao, Tongshuai Wang, Rui Wang, Xirong Guo, Junfen Fu

**Affiliations:** 1 Department of Endocrinology, Children’s Hospital, Zhejiang University School of Medicine, National Clinical Research Center for Child Health, Hangzhou, China; 2 Hongqiao International Institute of Medicine, Tongren Hospital, Shanghai Jiao Tong University School of Medicine, Shanghai, China; 3 Xinhua Hospital, Shanghai Jiao Tong University School of Medicine, Shanghai, China

Correction to: *Pediatric Research* 10.1038/s41390-025-04155-1, published online 10 June 2025

In this article the affiliation 7 details for Authors Bowen Zhu, Shumin Zhan, Hui Shi were incorrectly assigned.

The following has been aded: “Bowen Zhu, Shumin Zhan, and Hui Shi contributed equally to this work.”

The original artcle as been corrected.

