# Supplementary material for: Comparative analysis of adiposity indices for predicting 2-year hypertension incidence in children and adolescents: a retrospective study
Source: Pediatr Res. 2025 Jun 10;99(2):557–66. doi: 10.1038/s41390-025-04155-1 (PMC12956574; doi:10.1038/s41390-025-04155-1)
Supplement: Supplementary file 1 — Supplementary information [file 41390_2025_4155_MOESM1_ESM.pdf]

Supplemental data

Supplemental Figure S1. Flowchart of the participants selection. (A) Flowchart showing the study population selection process and showing the number of subjects excluded owing to different reasons; (B) Distribution of baseline (in 2009 year) and follow-up (in 2011 year) Systolic BP and Diastolic BP.

Supplemental Figure S2. Pearson correlation of anthropometric indices/ metabolic measures with continuous systolic BP (A) and diastolic BP (B).

Supplemental Table S1. Characteristics of participants included in the analysis and those excluded from the final analysis

Supplemental Table S2. Association between anthropometric indices/metabolic measures and hypertension diagnosis classfied by gender

Supplemental Table S3. Association between anthropometric indices/metabolic measures and hypertension diagnosis classfied by age

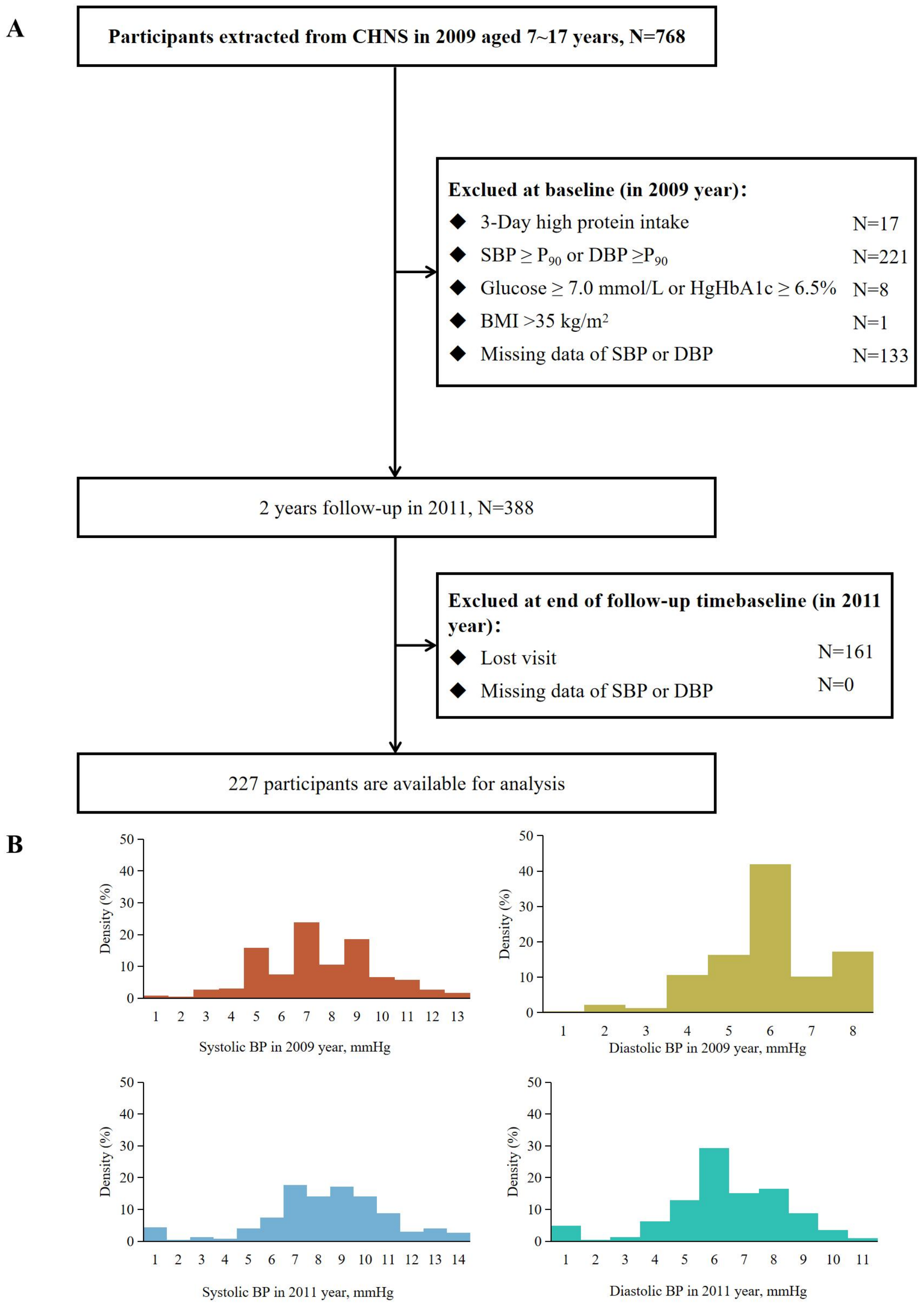

Supplemental Figure S1. Flowchart of the participants selection. (A) Flowchart showing the study population selection process and showing the number of subjects excluded owing to different reasons; (B) Distribution of baseline (in 2009 year) and follow-up (in 2011 year) Systolic BP and Diastolic BP.

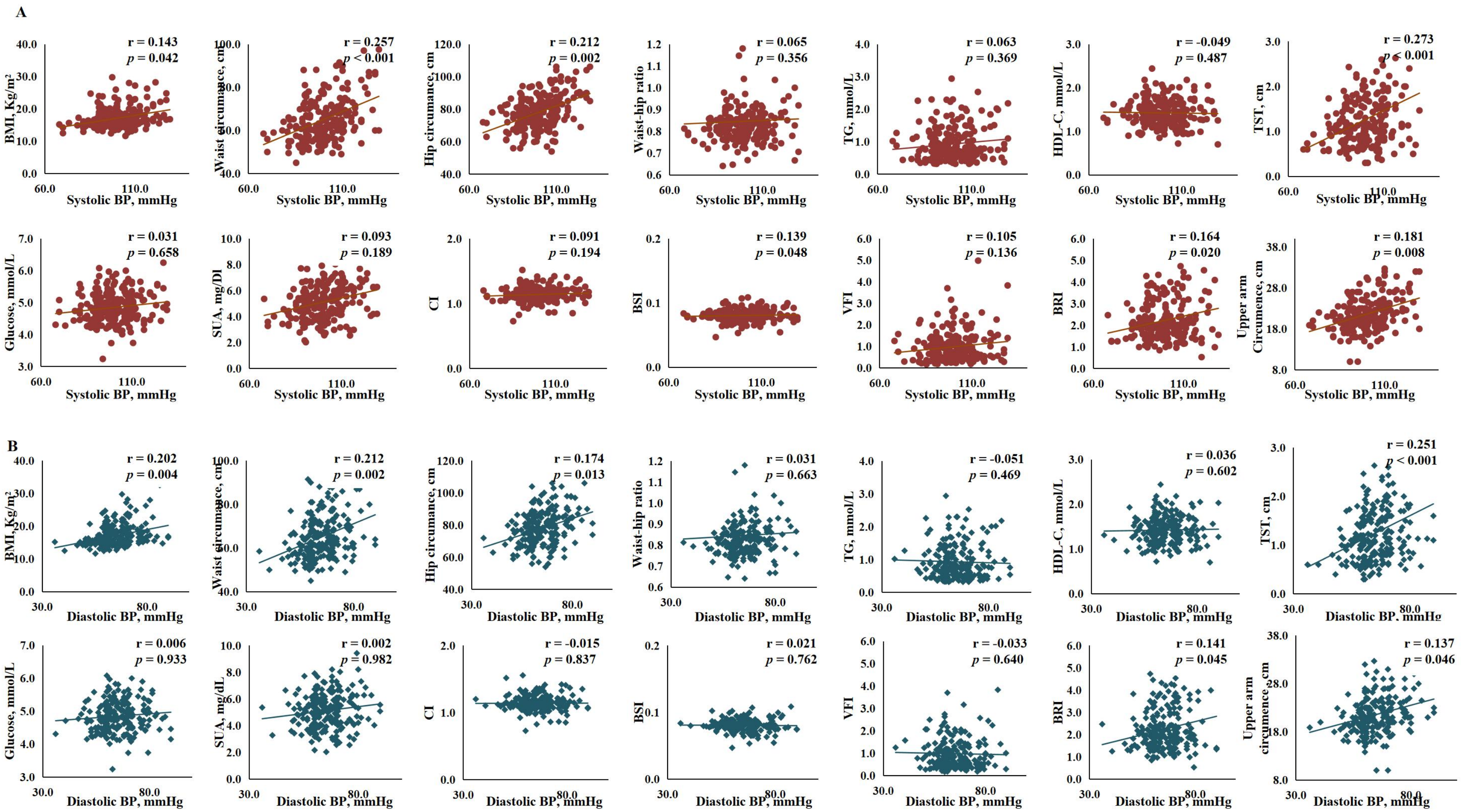

Supplemental Figure S2. Pearson correlation of anthropometric indices/metabolic measures with continuous systolic BP (A) and diastolic BP (B)

Supplemental Table S1. Characteristics of participants included in the analysis and those excluded from the final analysis

	Analytic Sample	Missing sample	*P*-value*
Participants (n)	227	161	
Demographics			
Age (years)	11.7 (2.3)	13.0 (2.5)	<0.001
Male (%)	147 (64.8)	113 (70.2)	0.263
Rural (%)	175 (77.1)	131 (81.4)	0.310
Anthropometry parameters			
Waist circumference (cm)	65.2 (10.4)	63.7 (8.1)	0.121
Hip circumference (cm)	78.2 (11.2)	77.2 (9.8)	0.363
WHR	0.83 (0.09)	0.83 (0.07)	0.727
BMI (kg/m^2)	17.1 (3.3)	17.6 (3.0)	0.128
Overweight (> P $_{75}$)	25 (11.0)	18 (11.2)	0.959
Riceps Skinfold Thickness (TST, cm)	1.1 (0.8-1.6)	1.03 (0.8-1.47)	0.200
Upper Arm Circumference (cm)	21.7 (4.4)	20.5 (3.7)	0.006
Systolic BP (mm Hg)	93.9 (11.5)	94.2 (10.9)	0.782
Diastolic BP (mm Hg)	61.1 (7.1)	61.8 (6.9)	0.338
Blood tests			
Albumin, g/L	48.3 (3.2)	49.3 (3.5)	0.005
Alanine Transaminase, U/L	14.0 (11.0-17.0)	14.0 (10.0-18.0)	0.910
Apolipoprotein B, mg/dL	0.6 (0.5-0.7)	0.6 (0.5-0.7)	0.504
Apolipoprotein A-1, mg/dL	0.97 (0.86-1.09)	0.98 (0.86-1.13)	0.663
Ferritin, ng/ml	43.87 (28.55-59.33)	44.53 (31.91-64.44)	0.334
Serum magnesium, mmol/L	0.95 (0.08)	0.93 (0.09)	0.009
Transferrin, mg/gL	294.0 (265.0-325.0)	291.0 (267.0-321.0)	0.295
Soluble Transferrin receptor, mg/L	1.5 (1.3-1.9)	1.6 (1.4-1.8)	0.684
Total protein, g/L	76.3 (4.9)	77.4 (4.7)	0.031
Serum uric acid, mg/dL	5.1 (1.3)	5.2 (1.4)	0.637
Total cholesterol, mmol/L	3.9 (0.6)	3.8 (0.7)	0.559
HDL cholesterol, mmol/L	1.4 (1.2-1.6)	1.4 (1.2-1.6)	0.730
LDL cholesterol, mmol/L	2.2 (1.8-2.6)	2.0 (1.7-2.4)	0.060
Triglycerides, mg/dL	0.8 (0.5-1.2)	0.8 (0.6-1.2)	0.642
eGFR, ml/ min/l.73m^2	81.6 (10.9)	77.0 (10.0)	<0.001
HbA1c, %	5.0 (0.4)	5.0 (0.4)	0.972
Blood Glucose, mmol/L	5.0 (0.5)	5.0 (0.5)	0.373
Creatinine, mg/dL	67.0 (61.0-74.0)	70.0 (65.0-77.0)	0.001
Anthropometric indices			
CI	1.13 (1.08-1.19)	1.14 (1.08-1.21)	0.575
BSI	0.08 (0.08-0.09)	0.08 (0.07-0.08)	<0.001
VFI	0.74 (0.44-1.25)	0.68 (0.44-1.18)	0.558
BRI	1.99 (1.55-2.70)	1.99 (1.63-2.54)	0.982

Health-related behavior			
Soft/Sugared fruit drinks	200 (88.5)	141 (88.1)	0.910
Total protein intake (g/day)	50.2 (39.7-64.8)	52.5 (41.3-64.7)	0.365
Total fat intake (g/day)	53.3 (35.7-71.6)	51.0 (32.5-69.5)	0.489
Total carbohydrate intake (g/day)	234.2 (191.6-270.2)	245.5 (195.3-305.3)	0.073
Total energy intake (kcal/day)	1646.0 (1327.2-1943.8)	1646.5 (1384.5-2057.7)	0.434

Abbreviation: BMI, body mass index; BP, blood pressure; BSI, body shape index; BRI, body roundness index; CI, conicity inex; eGFR, estimated glomerular filtration rate; HDL, high-density lipoprotein; HbA1c: Hemoglobin A1C; LDL, low-density lipoprotein; VFI, visceral fat index; WHR, waist to hip circumference ratio. Data are presented as No. (%), mean± SD; **P* values were calculated by using T test or Wilcoxon test for continuous variables and $\chi 2$ test for categorical variables.

Supplemental Table S2. Association between anthropometric indices/metabolic measures and hypertension diagnosis classfied by gender

	Boys				Girls			
	Model 1		Model 2		Model 1		Model 2	
	RR (95% CI)	*P* value	RR (95% CI)	*P* value	RR (95% CI)	*P* value	RR (95% CI)	*P* value
Hypertension diagnosis								
BMI, per SD increase, kg/m^2	1.25 (0.80-1.96)	0.335	1.31 (0.80-2.15)	0.291	1.93 (1.07-3.48)	0.029	2.23 (1.12-4.45)	0.023
Overweight (BMI>P_{75})	1.18 (0.33-4.23)	0.922	1.07 (0.28-4.08)	0.134	3.13 (0.67-14.62)	0.687	3.03 (0.58-15.8)	0.187
Waist circumference, per SD increase, cm	1.41 (0.91-2.18)	0.121	1.61 (0.99-2.61)	0.055	1.67 (0.90-3.10)	0.107	1.87 (0.95-3.69)	0.072
Central Obesity (Waist circumference >P_{75})	1.32 (0.45-3.86)	0.616	1.53 (0.50-4.74)	0.458	3.15 (0.93-10.69)	0.066	2.83 (0.80-10.03)	0.107
Hip circumference, per SD increase, cm	1.46 (0.88-2.45)	0.146	1.04 (0.99-1.10)	0.119	1.61 (0.90-2.88)	0.111	1.06 (0.99-1.12)	0.080
WHR, per SD increase	1.08 (0.74-1.56)	0.706	1.22 (0.81-1.85)	0.343	0.95 (0.81-1.11)	0.514	0.95 (0.80-1.12)	0.536
Triceps Skinfold Thickness (TST, cm）	1.47 (0.98-2.20)	0.063	1.77 (1.05-2.99)	0.032	2.46 (1.10-5.50)	0.028	2.97 (1.24-7.14)	0.015
Upper Arm Circumference (cm)	1.75 (1.04-2.94)	0.035	2.32 (1.21-4.47)	0.012	0.72 (0.42-1.22)	0.221	0.75 (0.43-1.29)	0.296
Triglycerides, per SD increase, mmol/L	1.19 (0.83-1.71)	0.348	1.08 (0.75-1.57)	0.674	0.82 (0.46-1.46)	0.499	0.86 (0.48-1.55)	0.625
Triglycerides ≥ 1.24, mmol/L	3.60 (1.28-10.16)	0.015	3.59 (1.13-11.43)	0.030	0.42 (0.14-1.28)	0.128	0.46 (0.15-1.44)	0.182
HDL cholesterol, per SD increase, mmol/L	0.99 (0.66-1.48)	0.941	1.20 (0.76-1.92)	0.435	0.93 (0.57-1.52)	0.433	0.91 (0.55-1.52)	0.727
HDL cholesterol≤1.03, mmol/L	1.90 (0.48-7.49)	0.362	1.40 (0.33-6.05)	0.649	2.16 (0.32-14.77)	0.467	2.23 (0.32-15.6)	0.422
Glucose, per SD increase, mmol/L	1.03 (0.68-1.57)	0.891	1.08 (0.68-1.74)	0.739	0.73 (0.43-1.26)	0.260	0.63 (0.35-1.14)	0.129
Glucose ≥ 5.6, mmol/L	1.81 (0.44-7.44)	0.412	1.44 (0.31-6.68)	0.640	N.A		N.A	
SUA (as continuous), mg/dL	1.00 (1.00-1.01)	0.580	1.00 (0.99-1.01)	0.602	1.00 (0.99-1.01)	0.577	1.00 (0.99-1.01)	0.856
SUA≥ P_{75}	1.14 (0.81-1.60)	0.442	1.16 (0.78-1.74)	0.470	0.93 (0.63-1.36)	0.693	1.00 (0.65-1.55)	0.986
Anthropometric indices								
CI, ln transformed	0.91 (0.56-1.47)	0.686	1.02 (0.61-1.70)	0.940	1.01 (0.67-1.53)	0.965	0.99 (0.64-1.53)	0.955
BSI, ln transformed	1.23 (0.75-2.01)	0.421	1.38 (0.81-2.36)	0.242	0.99 (0.66-1.49)	0.955	0.96 (0.62-1.48)	0.849
VFI, ln transformed	1.20 (0.83-1.72)	0.342	1.05 (0.71-1.54)	0.819	0.93 (0.53-1.61)	0.780	0.97 (0.56-1.70)	0.918

BRI, ln transformed	1.37 (0.50-3.77)	0.545	1.69 (0.58-4.91)	0.333	3.02 (0.80-11.31)	0.102	3.14 (0.79-12.4)	0.103
Elevated Systolic BP diagnosis								
BMI, per SD increase, kg/m^2	2.33 (1.11-4.89)	0.026	3.31 (1.24-8.82)	0.017	0.73 (0.15-3.58)	0.320	1.21 (0.27-5.46)	0.806
Overweight (BMI>P_{75})	5.02 (0.90-27.98)	0.066	3.75 (0.55-25.65)	0.179	3.35 (0.23-49.86)	0.381	17.68 (0.45-697.7)	0.126
Waist circumference, per SD increase, cm	3.39 (1.44-8.00)	0.005	9.53 (1.57-57.8)	0.014	1.91 (0.44-8.38)	0.392	3.85 (0.48-31.15)	0.206
Central Obesity (Waist circumference >P_{75})	11.27 (1.90-66.96)	0.008	30.43 (2.08-445.85)	0.013	6.14 (0.34-110.43)	0.219	11.32 (0.24-542.82)	0.219
Hip circumference, per SD increase, cm	1.88 (0.72-4.89)	0.199	2.83 (0.91-8.75)	0.072	2.36 (0.49-11.51)	0.287	9.08 (0.81-101.4)	0.073
WHR, per SD increase	1.71 (1.05-2.81)	0.033	2.55 (1.23-5.29)	0.012	0.87 (0.26-2.95)	0.825	0.50 (0.06-4.33)	0.525
Triceps Skinfold Thickness (TST, cm）	2.35 (1.19-4.64)	0.014	3.20 (1.14-8.95)	0.027	1.07 (0.22-5.10)	0.935	4.84 (0.29-81.11)	0.273
Upper Arm Circumference (cm)	2.96 (1.19-7.39)	0.020	9.65 (1.56-59.88)	0.015	1.03 (0.26-4.03)	0.966	1.24 (0.40-3.86)	0.706
Triglycerides, per SD increase, mmol/L	1.25 (0.62-2.53)	0.539	1.31 (0.71-2.41)	0.396	0.27 (0.03-2.52)	0.252	0.13 (0.01-2.08)	0.151
Triglycerides ≥ 1.24, mmol/L	2.23 (0.40-12.41)	0.362	3.88 (0.40-37.41)	0.242	N.A		N.A	
HDL cholesterol, per SD increase, mmol/L	1.13 (0.60-2.11)	0.705	1.20 (0.49-2.97)	0.690	0.48 (0.1-2.37)	0.371	0.62 (0.11-3.37)	0.579
HDL cholesterol≤1.03, mmol/L	1.22 (0.09-17.00)	0.881	0.85 (0.02-33.74)	0.933	N.A		N.A	
Glucose, per SD increase, mmol/L	1.42 (0.66-3.05)	0.367	2.62 (0.79-8.69)	0.117	0.66 (0.13-3.27)	0.609	0.34 (0.05-2.44)	0.286
Glucose ≥ 5.6, mmol/L	0.65 (0.05-7.97)	0.732	N.A		N.A		N.A	
SUA, per SD increase, mg/dL	1.00 (0.99-1.02)	0.848	1.01 (0.99-1.02)	0.249	0.99 (0.97-1.02)	0.665	1.00 (0.96-1.03)	0.760
SUA≥ P_{75}	1.03 (0.56-1.90)	0.932	1.51 (0.73-3.12)	0.271	1.07 (0.39-2.98)	0.896	1.14 (0.34-3.88)	0.830
Anthropometric indices								
CI, ln transformed	2.14 (0.90-5.11)	0.087	2.78 (1.05-7.38)	0.040	1.69 (0.60-4.72)	0.320	1.96 (0.54-7.20)	0.309
BSI, ln transformed	2.11 (0.91-4.92)	0.084	2.60 (0.90-7.40)	0.076	1.64 (0.52-5.11)	0.397	1.59 (0.39-6.46)	0.520
VFI, ln transformed	1.26 (0.64-2.47)	0.508	1.23 (0.64-2.36)	0.530	0.43 (0.04-4.26)	0.474	0.25 (0.03-2.08)	0.199
BRI, ln transformed	12.28 (1.68-89.98)	0.014	34.17 (1.87-624.57)	0.017	1.43 (0.04-46.68)	0.842	2.56 (0.04-149.89)	0.651
Elevated Diastolic BP diagnosis								
BMI, per SD increase, kg/m^2	1.22 (0.80-1.85)	0.353	1.20 (0.75-1.90)	0.448	1.78 (1.03-3.08)	0.041	2.15 (1.11-4.19)	0.024
Overweight (BMI>P_{75})	1.10 (0.31-3.91)	0.886	0.99 (0.26-3.79)	0.992	2.98 (0.65-13.56)	0.159	2.97 (0.58-15.2)	0.191

Waist circumference, per SD increase, cm	1.42 (0.94-2.14)	0.094	1.54 (0.97-2.45)	0.066	1.65 (0.89-3.07)	0.11	1.87 (0.95-3.70)	0.071
Central Obesity (Waist circumference >P_{75})	1.08 (0.37-3.16)	0.890	1.20 (0.38-3.75)	0.758	3.24 (0.96-10.89)	0.057	2.81 (0.80-9.83)	0.107
Hip circumference, per SD increase, cm	1.55 (0.96-2.50)	0.073	1.59 (0.91-2.77)	0.104	1.45 (0.84-2.52)	0.185	1.73 (0.90-3.36)	0.103
WHR, per SD increase	1.06 (0.72-1.50)	0.779	1.21 (0.79-1.86)	0.386	0.96 (0.83-1.12)	0.624	0.96 (0.81-1.12)	0.585
Triceps Skinfold Thickness (TST, cm）	1.27 (0.89-1.83)	0.192	1.37 (0.90-2.08)	0.142	1.78 (0.91-3.48)	0.093	2.36 (1.09-5.12)	0.030
Upper Arm Circumference (cm)	1.71 (1.05-2.79)	0.031	1.96 (1.06-3.63)	0.032	0.70 (0.42-1.18)	0.184	0.75 (0.43-1.29)	0.293
Triglycerides, per SD increase, mmol/L	1.14 (0.80-1.64)	0.469	0.98 (0.67-1.44)	0.922	0.81 (0.46-1.42)	0.455	0.86 (0.48-1.55)	0.621
Triglycerides ≥ 1.24, mmol/L	3.25 (1.19-8.84)	0.021	2.63 (0.84-8.30)	0.098	0.42 (0.14-1.26)	0.122	0.46 (0.15-1.44)	0.183
HDL cholesterol, per SD increase, mmol/L	1.00 (0.66-1.50)	0.991	1.25 (0.78-1.98)	0.351	0.92 (0.56-1.50)	0.731	0.91 (0.55-1.52)	0.723
HDL cholesterol≤1.03, mmol/L	1.64 (0.46-5.85)	0.443	1.24 (0.32-4.84)	0.761	1.98 (0.29-13.34)	0.485	2.17 (0.31-15.09)	0.433
Glucose, per SD increase, mmol/L	1.02 (0.67-1.55)	0.934	1.03 (0.65-1.65)	0.893	0.73 (0.43-1.25)	0.250	0.63 (0.35-1.14)	0.128
Glucose ≥ 5.6, mmol/L	2.42 (0.63-9.30)	0.173	1.76 (0.4-7.79)	0.458	N.A		N.A	
SUA, per SD increase, mg/dL	1.00 (1.00-1.01)	0.217	1.00 (1.00-1.01)	0.460	1.00 (0.99-1.01)	0.502	1.00 (0.99-1.01)	0.846
SUA≥ P_{75}	1.26 (0.92-1.74)	0.151	1.21 (0.81-1.81)	0.363	0.91 (0.62-1.33)	0.623	1.00 (0.65-1.54)	0.994
Anthropometric indices								
CI, per SD increase	0.96 (0.60-1.54)	0.877	1.07 (0.65-1.76)	0.802	1.04 (0.69-1.56)	0.858	1.00 (0.65-1.52)	0.982
BSI, per SD increase	1.25 (0.77-2.03)	0.368	1.45 (0.85-2.47)	0.171	1.02 (0.68-1.52)	0.939	0.97 (0.63-1.48)	0.875
VFI, per SD increase	1.18 (0.83-1.68)	0.363	0.98 (0.67-1.44)	0.932	0.91 (0.53-1.56)	0.723	0.97 (0.55-1.69)	0.911
BRI, per SD increase	1.28 (0.47-3.50)	0.627	1.59 (0.56-4.54)	0.385	2.98 (0.80-11.13)	0.104	3.15 (0.8-12.43)	0.102

Abbreviation: BMI, body mass index; BP, blood pressure; BSI, body shape index; BRI, body roundness index; CI, conicity inex; eGFR, estimated glomerular filtration rate; HDL, high-density lipoprotein; HbA1c: Hemoglobin A1C; LDL, low-density lipoprotein; VFI, visceral fat index; WHR, waist to hip circumference ratio. Model 1 adjusted for baseline Systolic BP and/or Diastolic BP; Model 2 adjusted for baseline residence, eGFR, soft fruit drinks, and total carbohydrate intake, in addition to the covariate in model 1.

Supplemental Table S3. Association between anthropometric indices/metabolic measures and hypertension diagnosis classfied by age

	7~12 years				13~17 years			
	Model 1		Model 2		Model 1		Model 2	
	RR (95% CI)	*P* value	RR (95% CI)	*P* value	RR (95% CI)	*P* value	RR (95% CI)	*P* value
Hypertension diagnosis								
BMI, per SD increase, kg/m^2	2.25 (1.41-3.57)	<0.001	2.33 (1.38-3.93)	0.002	0.99 (0.61-1.62)	0.975	1.13 (0.62-2.04)	0.693
Overweight (BMI>P_{75})	3.36 (1.17-9.63)	0.024	3.25 (0.98-10.80)	0.054	0.61 (0.10-3.71)	0.591	0.60 (0.08-4.61)	0.624
Waist circumference, per SD increase, cm	2.38 (1.46-3.87)	<0.001	2.42 (1.42-4.12)	0.001	1.06 (0.65-1.73)	0.817	1.19 (0.68-2.07)	0.538
Central Obesity (Waist circumference >P_{75})	4.39 (1.72-11.2)	0.002	3.90 (1.36-11.18)	0.012	1.08 (0.31-3.72)	0.904	1.25 (0.34-4.61)	0.735
Hip circumference, per SD increase, cm	1.07 (1.02-1.12)	0.003	2.01 (1.17-3.44)	0.011	1.01 (0.95-1.07)	0.705	1.28 (0.57-2.84)	0.553
WHR, per SD increase	1.00 (0.87-1.16)	0.984	0.99 (0.86-1.14)	0.878	1.03 (0.70-1.51)	0.886	1.06 (0.72-1.58)	0.760
Riceps Skinfold Thickness (TST, cm）	2.48 (1.51-4.08)	<0.001	2.67 (1.50-4.75)	<0.001	1.12 (0.68-1.83)	0.659	1.25 (0.70-2.26)	0.451
Upper Arm Circumference (cm)	1.25 (0.84-1.85)	0.272	1.29 (0.87-1.92)	0.208	0.9 (0.44-1.81)	0.760	1.04 (0.45-2.39)	0.922
Triglycerides, per SD increase, mmol/L	1.02 (0.71-1.45)	0.934	0.92 (0.61-1.40)	0.708	1.20 (0.70-2.03)	0.512	1.31 (0.73-2.33)	0.363
Triglycerides ≥ 1.24, mmol/L	1.12 (0.42-2.99)	0.815	1.01 (0.34-2.99)	0.989	1.83 (0.63-5.30)	0.264	2.11 (0.65-6.84)	0.216
HDL cholesterol, per SD increase, mmol/L	1.22 (0.81-1.82)	0.341	1.36 (0.86-2.16)	0.187	0.76 (0.46-1.24)	0.268	0.67 (0.39-1.15)	0.142
HDL cholesterol≤1.03, mmol/L	0.60 (0.12-3.02)	0.536	0.55 (0.10-3.05)	0.496	2.46 (0.61-9.96)	0.206	3.22 (0.69-15.12)	0.138
Glucose, per SD increase, mmol/L	0.85 (0.57-1.26)	0.412	0.91 (0.59-1.41)	0.679	0.95 (0.57-1.57)	0.835	0.96 (0.52-1.78)	0.906
Glucose ≥ 5.6, mmol/L	0.73 (0.08-6.65)	0.777	0.96 (0.10-9.63)	0.975	1.17 (0.25-5.51)	0.839	1.05 (0.18-6.16)	0.957
SUA (as continuous), mg/dL	1.00 (0.99-1.01)	0.992	1.00 (0.99-1.01)	0.783	1.00 (0.99-1.01)	0.497	1.00 (0.99-1.01)	0.818
SUA≥ P_{75}	1.01 (0.74-1.40)	0.932	1.08 (0.73-1.59)	0.699	0.95 (0.64-1.40)	0.782	1.02 (0.64-1.62)	0.927
Anthropometric indices								
CI, ln transformed	1.13 (0.77-1.67)	0.933	1.09 (0.72-1.66)	0.672	1.03 (0.62-1.70)	0.914	1.04 (0.62-1.75)	0.879
BSI, ln transformed	1.21 (0.82-1.77)	0.338	1.21 (0.80-1.81)	0.371	1.15 (0.66-2.02)	0.622	1.14 (0.62-2.09)	0.667
VFI, ln transformed	1.12 (0.81-1.55)	0.495	0.95 (0.64-1.41)	0.808	1.41 (0.78-2.56)	0.259	1.41 (0.72-2.78)	0.318

BRI, ln transformed	6.28 (2.06-19.11)	0.001	6.12 (1.80-20.78)	0.004	0.93 (0.31-2.78)	0.892	1.05 (0.32-3.51)	0.934
Elevated Systolic BP diagnosis								
BMI, per SD increase, kg/m^2	11.94 (1.12-126.75)	0.040	N.A		1.14 (0.56-2.30)	0.719	1.73 (0.70-4.20)	0.233
Overweight (BMI>P_{75})	30.08 (1.88-482.03)	0.016	N.A		0.88 (0.07-10.73)	0.917	2.60 (0.10-69.04)	0.569
Waist circumference, per SD increase, cm	N.A		N.A		1.21 (0.54-2.68)	0.646	2.11 (0.77-5.77)	0.148
Central Obesity (Waist circumference >P_{75})	N.A		N.A		2.33 (0.40-13.64)	0.348	4.13 (0.54-31.79)	0.173
Hip circumference, per SD increase, cm	N.A		N.A		0.62 (0.20-1.86)	0.389	0.86 (0.24-3.15)	0.822
WHR, per SD increase	1.23 (0.86-1.74)	0.254	1.64 (0.91-2.9)	0.101	1.46 (0.85-2.50)	0.169	1.57 (0.91-2.70)	0.106
Riceps Skinfold Thickness (TST, cm）	N.A		N.A		1.05 (0.51-2.18)	0.887	1.63 (0.66-4.01)	0.287
Upper Arm Circumference (cm)	4.93 (1.14-21.41)	0.033	N.A		0.87 (0.29-2.65)	0.811	2.54 (0.52-12.57)	0.252
Triglycerides, per SD increase, mmol/L	1.16 (0.24-5.47)	0.855	0.16 (0-19.75)	0.452	1.10 (0.46-2.64)	0.831	1.20 (0.45-3.21)	0.717
Triglycerides ≥ 1.24, mmol/L	2.95 (0.19-45.57)	0.438	N.A		1.00 (0.16-6.13)	0.998	1.33 (0.18-10.13)	0.783
HDL cholesterol, per SD increase, mmol/L	0.21 (0.04-1.27)	0.089	N.A		1.27 (0.62-2.59)	0.510	1.19 (0.55-2.55)	0.659
HDL cholesterol≤1.03, mmol/L	N.A		N.A		1.3 (0.12-14.31)	0.829	2.02 (0.12-34.01)	0.627
Glucose, per SD increase, mmol/L	1.6 (0.61-4.22)	0.338	N.A		1.17 (0.51-2.69)	0.716	0.58 (0.13-2.48)	0.457
Glucose ≥ 5.6, mmol/L	N.A		N.A		1.00 (0.09-11.22)	0.998	N.A	
SUA, per SD increase, mg/dL	1.02 (1-1.04)	0.034	1.13 (0.96-1.34)	0.143	0.99 (0.97-1.00)	0.102	0.99 (0.97-1.01)	0.258
SUA≥ P_{75}	2.9 (0.99-8.55)	0.053	N.A		0.66 (0.33-1.33)	0.247	0.73 (0.31-1.73)	0.477
Anthropometric indices								
CI, ln transformed	8.49 (1.31-54.91)	0.025	N.A		1.07 (0.45-2.50)	0.884	1.32 (0.55-3.17)	0.532
BSI, ln transformed	9.91 (1.09-90)	0.042	12.07 (0.2-738.05)	0.235	1.11 (0.45-2.76)	0.819	1.35 (0.45-4.03)	0.591
VFI, ln transformed	1.49 (0.62-3.55)	0.372	0.82 (0.04-18.48)	0.901	1.22 (0.53-2.81)	0.639	1.15 (0.47-2.81)	0.765
BRI, ln transformed	N.A		N.A		1.13 (0.19-6.63)	0.897	3.3 (0.35-31.23)	0.298
Elevated Diastolic BP diagnosis								
BMI, per SD increase, kg/m^2	2.05 (1.31-3.22)	0.002	2.15 (1.27-3.65)	0.004	0.99 (0.61-1.6)	0.954	1.09 (0.60-1.99)	0.770
Overweight (BMI>P_{75})	2.79 (0.96-8.13)	0.061	2.92 (0.84-10.22)	0.093	0.75 (0.13-4.36)	0.745	0.85 (0.11-6.81)	0.877

Waist circumference, per SD increase, cm	2.19 (1.36-3.54)	0.001	2.19 (1.29-3.73)	0.004	1.01 (0.62-1.63)	0.982	1.13 (0.64-1.99)	0.665
Central Obesity (Waist circumference >P_{75})	3.83 (1.48-9.89)	0.006	3.61 (1.2-10.84)	0.022	0.79 (0.22-2.79)	0.714	0.99 (0.25-3.88)	0.982
Hip circumference, per SD increase, cm	1.88 (1.16-3.05)	0.011	1.70 (1.00-2.90)	0.050	1.20 (0.63-2.28)	0.586	1.39 (0.60-3.20)	0.440
WHR, per SD increase	1.00 (0.87-1.15)	0.976	0.99 (0.86-1.13)	0.828	0.96 (0.65-1.41)	0.830	1.00 (0.67-1.49)	0.997
Riceps Skinfold Thickness (TST, cm）	1.98 (1.28-3.05)	0.002	2.03 (1.24-3.31)	0.005	1.06 (0.65-1.72)	0.821	1.15 (0.63-2.07)	0.654
Upper Arm Circumference (cm)	0.86 (0.43-1.72)	0.500	1.18 (0.80-1.74)	0.411	1.14 (0.78-1.66)	0.668	0.95 (0.40-2.24)	0.905
Triglycerides, per SD increase, mmol/L	1.01 (0.71-1.46)	0.742	0.92 (0.59-1.43)	0.694	1.10 (0.65-1.80)	0.715	1.20 (0.66-2.18)	0.547
Triglycerides ≥ 1.24, mmol/L	1.15 (0.43-3.07)	0.718	0.99 (0.33-2.98)	0.989	1.55 (0.54-4.49)	0.418	1.70 (0.51-5.66)	0.391
HDL cholesterol, per SD increase, mmol/L	1.25 (0.84-1.86)	0.278	1.47 (0.92-2.35)	0.107	0.71 (0.43-1.18)	0.188	0.61 (0.35-1.08)	0.093
HDL cholesterol≤1.03, mmol/L	0.59 (0.12-2.99)	0.523	0.48 (0.09-2.71)	0.407	2.24 (0.59-8.51)	0.235	3.21 (0.70-14.81)	0.135
Glucose, per SD increase, mmol/L	0.80 (0.53-1.20)	0.275	0.86 (0.55-1.35)	0.510	0.94 (0.57-1.53)	0.795	1.00 (0.54-1.85)	0.995
Glucose ≥ 5.6, mmol/L	0.83 (0.09-7.59)	0.866	1.32 (0.13-13.72)	0.814	1.39 (0.31-6.24)	0.668	1.45 (0.24-8.72)	0.688
SUA, per SD increase, mg/dL	1.00 (0.99-1.01)	0.823	1.00 (0.99-1.01)	0.535	1.00 (0.99-1.01)	0.450	1.00 (0.99-1.01)	0.777
SUA≥ P_{75}	1.04 (0.75-1.44)	0.806	1.14 (0.77-1.68)	0.515	0.94 (0.64-1.37)	0.735	1.01 (0.64-1.62)	0.957
Anthropometric indices								
CI, per SD increase	1.13 (0.77-1.68)	0.530	1.08 (0.71-1.65)	0.716	0.99 (0.60-1.63)	0.962	1.01 (0.60-1.71)	0.974
BSI, per SD increase	1.22 (0.83-1.79)	0.324	1.21 (0.79-1.83)	0.380	1.11 (0.63-1.93)	0.723	1.13 (0.61-2.09)	0.966
VFI, per SD increase	1.12 (0.81-1.55)	0.508	0.94 (0.62-1.43)	0.775	1.38 (0.77-2.47)	0.286	1.39 (0.69-2.77)	0.356
BRI, per SD increase	5.61 (1.85-16.98)	0.002	6.67 (1.83-24.33)	0.004	0.87 (0.29-2.59)	0.797	1.01 (0.30-3.44)	0.985

Abbreviation: BMI, body mass index; BP, blood pressure; BSI, body shape index; BRI, body roundness index; CI, conicity inex; eGFR, estimated glomerular filtration rate; HDL, high-density lipoprotein; HbA1c: Hemoglobin A1C; LDL, low-density lipoprotein; VFI, visceral fat index; WHR, waist to hip circumference ratio. Model 1 adjusted for baseline Systolic BP and/or Diastolic BP; Model 2 adjusted for baseline residence, eGFR, soft fruit drinks, and total carbohydrate intake, in addition to the covariate in model 1
